# Adaptive Software Architecture Based on Confident HCI for the Deployment of Sensitive Services in Smart Homes

**DOI:** 10.3390/s150407294

**Published:** 2015-03-25

**Authors:** Mario Vega-Barbas, Iván Pau, María Luisa Martín-Ruiz, Fernando Seoane

**Affiliations:** 1 School of Technology and Health, KTH-Royal Institute of Technology, Huddinge SE-14152, Sweden; E-Mail: fsm@kth.se (F.S.); 2 T>SIC Research Group, ETSIST, Universidad Politécnica de Madrid, Ctra. Valencia Km. 7, Madrid 28038, Spain; E-Mails: ipau@diatel.upm.es (I.P.); marisam@diatel.upm.es (M.L.M.-R.); 3 School of Engineering, University of Borås, SE-50190 Borås, Sweden

**Keywords:** telemedicine sensor software architecture, assistive services, digital home, activity centered design, confidence

## Abstract

Smart spaces foster the development of natural and appropriate forms of human-computer interaction by taking advantage of home customization. The interaction potential of the Smart Home, which is a special type of smart space, is of particular interest in fields in which the acceptance of new technologies is limited and restrictive. The integration of smart home design patterns with sensitive solutions can increase user acceptance. In this paper, we present the main challenges that have been identified in the literature for the successful deployment of sensitive services (e.g., telemedicine and assistive services) in smart spaces and a software architecture that models the functionalities of a Smart Home platform that are required to maintain and support such sensitive services. This architecture emphasizes user interaction as a key concept to facilitate the acceptance of sensitive services by end-users and utilizes activity theory to support its innovative design. The application of activity theory to the architecture eases the handling of novel concepts, such as understanding of the system by patients at home or the affordability of assistive services. Finally, we provide a proof-of-concept implementation of the architecture and compare the results with other architectures from the literature.

## 1. Introduction

Ubiquitous computing presents an interesting scientific practice in which the computing capacity increases significantly due to the large number of technological elements that are integrated seamlessly into the environment and our daily lives. The Internet of Things (IoT) represents the next step in this concept and offers the opportunity to arrange these physical objects into communication-actuating networks in a way that is similar to how digital information is organized on the Internet [1,2]. In this way, both concepts in conjunction with ambient intelligence form a novel scenario that provides a customized view of the physical world [3] and thus offers interesting options to develop new interactive services for assisting people in performing certain tasks. 

The interaction model that this scenario offers is of particular interest in the definition of smart spaces that are used for the deployment of critical and sensible services, such as the promotion of personal autonomy or telemedicine and e-health solutions [4]. In particular, this scenario is being studied as a new concept of development in the field of Ambient Assisted Living (AAL) and homecare because it facilitates the care of patients in their usual environments [5]. It also provides socio-economic benefits that must be taken into account, such as the promotion of user autonomy, reduction of costs in the management of chronic patients and the possibility of establishing a more direct relationship between primary and specialized care [6].

However, although the current trends in health care and the actual needs of society clearly justify the inclusion of pervasive technology and wireless sensor networks in the development of new types of personalized healthcare applications and assistive services [7,8], unresolved issues and challenges prevent the final implementation and adoption of such pervasive healthcare solutions. The sensors and actuators that are used in smart spaces represent a vague and strange concept that hinders their acceptance by final users. Researchers often encounter serious complications when attempting to deploy this technology in real homes, which is the *location of choice* for the implementation of assistive environments, mainly due to the difficulty of residents in understanding its operation and internal behavior [9,10]. In addition, when assistive environments are applied to clinical practice, strict medical and safety standards must be met, and the services must be administered often and always be supervised by medical personnel, such as doctors, nurses, and therapists, who are commonly referred to as caregivers. These caregivers, as well as the other users, usually have limited knowledge of the technology involved; therefore, the implemented systems must be simple, intuitive and effective. Finally, the integrators that install the devices and certify their proper functioning often encounter difficulties when trying to use components from different manufacturers that utilize proprietary communication protocols.

The Smart Home is a particularly interesting case of assistive environment. Many studies have defined the home as the suitable place to perform certain clinical treatments and assistive therapies [5,6]. Information and Communication Technology (ICT) can effectively facilitate and even enable novel applications. However, even at home, users may not feel comfortable or confident with these technologies, particularly in areas related to their own health [11]. Therefore, the proper integration of ICT and the home would allow taking advantage of the communication and automation capabilities of ICT with the customization and acceptance advantages of home environments. In this regard, the main objective of the smart home is the correct integration of technologies at home to facilitate the deployment of useful services and maximize user acceptance.

This paper presents a set of requirements for the future development of confident sensitive solutions at home using physical and daily smart objects as sensors. Because the requirements are not easy to fulfill for all services, we propose the development of a smart home platform to address most of the requirements and support the deployment and running of sensitive services. The smart home platform is thought of as middleware for services that is supported by heterogeneous sensors. In this way, the service designer can use the smart home platform as both a development platform, which allows focusing on the specific logic of the service, and a deployment platform that controls the life cycle of the service. 

We present a software architecture as a novel design method to support the transformation of common spaces into smart spaces. The architecture emphasizes user interaction as a key concept to facilitate the acceptance of sensitive services by the end users. In this sense, the functionality described in the architecture reflects the research on confident Human-Computer Interaction (HCI) that has been developed by the authors [11]. In addition, this software architecture is based on the theory of activity concepts in that all of the activities of the users are decomposed in actions and tasks [12,13]. Thus, the use of actions and tasks as design elements provides a new way to model smart spaces for assistive services, such as from a point of view that is similar to human behavior, to allow a better overall understanding of the implemented system. In this way, the Activity Centered Design method [14] provides a guideline for the software architecture to obtain an adaptive and robust solution. The research presented in this paper uses this concept to design the internal behavior of the software architecture and to model the interactions between users and the system.

This paper is organized as follows. After this introduction, the state-of-the-art of the software architectures that are related to this topic is presented and analyzed in Section 2. Section 3 describes the major challenges for HCI architectures in the context of telemedicine and assistive services. Such challenges were identified based on the analysis that was performed in the previous section and a systematic literature review. Section 4 describes the proposed software architecture, and Section 5 verifies the architecture with a case study. Finally, Section 6 and Section 7 discuss the results and the conclusions drawn from this study. 

## 2. Related Work 

The design and construction of software architectures for the deployment of sensible services, such as telemedicine and assistive solutions, have historically focused on both patients and the clinical environment close to home. However, these solutions pose problems of user acceptance due to the use of the home as the means of deployment. For example, studies such as [5] have shown that the actual use of such solutions for medical consultations is low.

From a computational point of view and as a result of the emergence of the ubiquitous computing paradigm, including the IoT and Ambient Intelligence, researchers have attempted to apply the advantages offered by the construction of smart spaces to clinical and assistive practice. This paper focuses on general architectures to support the deployment of heterogeneous services in the smart home. In the following sections, current solutions that are aligned with the objective of this paper are detailed.

### 2.1. Current Commercial Solutions

Nowadays the market is offering to advanced users several solutions based on the IoT concept to implement services related to visualization of health data, e.g., Apple Health, Google Fit, Fitbit, to easily develop connected applications, e.g., IFTTT, or to access to automate home activities, e.g., SmartThings. These solutions are focused on very specific user needs following the Do-it-Yourself (DIY) approach and are usually deployed on wearable, mobile, tablet or computer devices.

IFTTT (IFTTT Inc., San Diego, CA, USA) presents an action-reaction middleware that allows users to interconnect web services by chains of conditional statements. This technology enables users to capture changes in the state of web services and trigger their own applications according to those changes. Users need some knowledge about web environments and must be getting familiar with web applications in order to take advantage of this powerful solution. Like IFTTT, AppleHealth (Apple Inc., Cupertino, CA, USA) offers users an interaction solution to centralize health mobile applications oriented to manage their health information.

SmartThings (SmartThings Inc., Samsung, Washington, DC, USA) GrandCare Systems (GrandCare Systems, West Bend, WI, USA) and BeClose (BeClose, Vienna, VA, USA) are examples of complete IoT solutions. SmartThings offers final users to control and monitor their home from one usable mobile phone application. This home platform allows interconnecting several home elements such as furniture, doors and some electrical appliances by sensors and actuators (owner and third-party) in order to automate usual human tasks in a home. GrandCare System and BeClose are similar to SmartThings but strong oriented to the elderly home care. Both solutions try to reduce healthcare costs and improve healthcare outcomes by providing a hub point where all stakeholders of these services are involved.

### 2.2. ATLAS

The Mobile Computer Laboratory of the University of Florida has developed a scalable framework architecture for pervasive computing systems. This architecture allows for abstracting the sensors and actuators that are deployed to a smart home as services [15] from the points of view of developers and programmers. The architecture is oriented at integrators and suppliers, and the solutions that can be developed are limited to a particular type of sensor. Those sensors, which are developed by the same research group, cannot communicate with third party devices transparently. Moreover, this solution omits the computational process to the home users, who are the residents of the intelligent environment.

### 2.3. S3OiA and WSO_2_ Reference Architecture for the IoT

S3OiA [16] presents a research study that is intended to contribute to the standardization and interoperability of the future Internet through an open and scalable solution. It is a syntactic and semantic service-oriented architecture that allows the integration of any object or device, regardless of its nature, into the IoT. Additionally, the architecture enables the use of devices that are deployed in smart environments as a substrate for the automatic composition of complex applications through a triple paradigm semantic space. Thus, the creation of applications is dynamic and adaptive because they can evolve according to the context in which they are executed. The architecture also accounts for the possibility of dealing with different types of users that provide a suitable model of interaction based on their characteristics. However, its global approach, which is oriented towards the DIY concept, prevents it from offering solid solutions for security and reliability in telemedicine and e-Health applications.

Recently, WSO2 presented their reference architecture for the IoT [17]. This white paper introduces the requirements for interacting with and managing devices in the context of the IoT. The reference architecture is focused on architects and developers of IoT projects with the aim of providing them a starting point that covers the major requirements of IoT projects and systems, which include connectivity, device management, data processing, scalability and security. As with S3OiA, this reference architecture presents an overview of the needs of the IoT and attempts to provide a global solution for any type of service that is developed in the context of the IoT. However, it lacks the user’s point of view and prevents any requirement about user acceptance or user confidence, which is another key concept for the development of sensible services. 

### 2.4. Smart Daily Objects

Smart daily objects are a usable and understandable interface between people and the environment in which they operate. These elements provide contextual information, such as physical location, origin, and condition, while supporting a model of interaction that is familiar to the person who interacts with them. This requires virtually augmenting the daily objects using information technology and allowing integration into an intelligent environment to obtain the maximum benefit from joining the physical world with the digital world [18]. The goal of using this type of element as a means to create smart spaces is to ensure an optimal level of confidence and acceptance of ambient intelligence systems. Lopez-de-Armentia *et al.* [19] provide an example of how an everyday smart object, such as a coffee maker, can bring about social change towards energy efficiency.

The main problem with the use of these elements is the need for a high degree of design and development expertise, which excludes non-technical people. Although there are initiatives for the development of DIY platforms to solve this problem, the services that are developed in the field of telemedicine and e-health involve a high level of security and reliability.

### 2.5. Telemedicine and Assistive Experiences in the Digital Home

In [20], the authors present a web-based application for a consultation process solution. The authors claim that this work represents a correct solution from the points of view of usability and the number of derivations to primary care. However, this study lacks a detailed assessment of user satisfaction and potential acceptance.

The work presented in [21] provides a framework for user-centered design for conducting teleconsultation systems through appropriate communications channels between patients and doctors. This study focused on maximizing the acceptance of the service by users via an initial study of the ecosystem of users and technology acceptance models. The work described in [22] provides a series of recommendations for telemedicine services and e-Health to ensure the proper levels of usability and acceptance. In both cases, the solutions are based on a user-centered design that associates the sustainability of the system and the evolution of the user who is experiencing it; both parameters are highly dynamic and unpredictable.

Finally, in [23], the authors propose a complete solution that combines the concepts of ubiquitous computing and telemedicine. This solution uses an architecture that is based on the IoT to enter the patient environment in the clinical setting and provides a high level of connectivity between clinical devices (interoperability) and telemonitoring systems. However, this research is unclear as to the role that non-technical users should play in the solutions and how this work addresses the problem of final acceptance by those users.

### 2.6. UniversAAL 

The universAAL (uAAL) project attempts to define and produce an open platform to provide a standardized, reliable and economic approach to develop AAL solutions [24]. To achieve this, uAAL defines logical environments (AAL spaces) that are composed of embedded networked artifacts (software and hardware) that are oriented to a specific human user or a set of users. The core part of uAAL is the middleware, which ensures that all universAAL elements (uAAL nodes) in a space can interoperate. The logical execution of the middleware is organized as containers, and there are different containers depending on the type of device in which it is deployed. Thus, the middleware can run as OSGi bundles and Java applications in computers or embedded systems and as APKs in Android smartphones. The pairing communication between each node is performed by defining specific-purpose busses. Finally, a uAAL application is any piece of software that can run on the container and uses the uAAL buses to provide an AAL service.

This project defines a correct AAL solution for developers, but it is limited in the user-system interaction. uAAL uses common graphical user interfaces (GUIs) as interaction elements, and the architecture can only use artifacts that can integrate and develop GUIs. Additionally, uAAL requires introducing new technology into houses, such as smartphones, smart TVs, computers, and other elements, which might not be present in every home or be challenging for the elderly to use.

## 3. Architecture Challenges of the HCI for Telemedicine and Assistive Services at Home

The development of a telemedicine or assistive service that allows the integration of the different roles that are involved in clinical or healthcare practices in an effective, safe, intuitive and simple manner is a task that poses serious challenges [25,26,27,28,29]. The addition of sensing technologies, which are sometimes intrusive and can be incomprehensible to non-technical users, raises additional challenges from immersing users in highly sophisticated and often intimate environments such as smart spaces.

Within smart spaces, this paper focuses on the digital home. In an academic and industrial context, the home is considered as the appropriate environment for the deployment of telemedicine and assistive solutions [9] because one of the fundamental functions of this type of space is to enable the continuity of care, and the home is a natural space to accommodate this requirement.

A literature review of telemedicine solutions and technologies that are deployed at home shows that most new contributions have included adaptations of sensing technologies that are used in other applications. However, both sensitive services and the digital home have characteristics that significantly alter the requirements that are imposed on the technologies in other application areas. 

One of the fundamental issues in defining a solution is the proposal of a functional architecture. The architecture formally models the requirements, so the solutions should follow the patterns that are described in the architecture to fulfil those requirements. Moreover, the architecture must be adapted to all of the actors and users that are involved in the solution and take into account the evolution in the behavior of those actors. The proper choice of elements determines the architecture and, critically, the chances for the success and acceptance of the proposed solution over time.

A requirements gathering process is needed to define an appropriate architecture. Other authors have described several of these requirements [25,26,27]; however, novel requirements are only described for sophisticated environments and are not properly described and customized for telemedicine solutions at home and vice-versa. The novel requirements are as follows:

C1 *Installing devices.* As we presented in Section 2, current solutions enable easy device installation by users. However, these solutions lack the rigor that is required for medical applications, so technology integrators must be involved in most cases. Technology integrators must verify the proper operation of the deployment. Therefore, the architecture cannot ignore the role of the integrator.
C2 *Interacting with the user’s home.* The interaction pattern and the application logic are closely related in most applications. Thus, a change in the logic of the application implies changes in the user interface and *vice-versa*. However, this model does not fit well with telemedicine solutions in a digital home. Home users usually learn patterns of interaction and do not properly handle meaningful changes. The final design must disengage the strong connection between logic and the interaction pattern.
C3 *Evolution of the solution.* Any solution that is based on information and communication technologies should be considered an artifact that evolves with its users. The evolution must allow both the addition of new features to those that are already deployed and a change in the actual conduct of the application based on the acquisition of technical skills and confidence by the users [30].
C4 *Resilience.* The final use of a technology depends on the analysis and design for the solution, the intended use, and how the users decide to use it. There are cases of technologies that have been used successfully but in different ways that what was initially intended [30]. Thus, an architecture that is oriented to sensitive services must take into account both the user experience and possible variations of use of the technology.
C5 *User confidence*. A key aspect is the confidence that users can develop towards the solution. If this aspect is not accounted for, the acceptance of proposed solutions will be limited, especially in the case of real and existing alternatives that provide a similar service. This is considered as one of the main reasons for the limited impact of telemedicine solutions today [25]. The architecture must be able to respond to the fears of users that are trying to improve their confidence in the system. In this research, the term confidence is defined as an arrangement of two key human abilities, understanding and control [11].

While methods are available to develop solutions that take into account several of the challenges described above, reference architectures for defining design patterns for solutions that account for user confidence were not found. The definition of a robust architecture that accounts for these requirements may be a breakthrough for the development and deployment of telemedicine services that allow not only the effective use by one type of user at a given time but also by a wide range of users whose skills can evolve over time. Table 1 compares the challenges presented above with the set of system and service qualities that an assistive system should possess according to Becker [31].

**Table 1:** Challenges of telemedicine architectures *vs*. Becker’s assistive requirements.

Challenges	Affordability	Usability and User Exp.	Suitability	Dependability	Adaptivity	Extensiblity	Resource Efficiency	Heterogeneity
**(1)**	✓	✓	✓	-	✓	✓	✓	✓
**(2)**	✓	✓	✓	✓	-	-	-	-
**(3)**	✓	✓	-	-	✓	✓	✓	✓
**(4)**	✓	✓	✓	✓	✓	-	-	-
**(5)**	✓	✓	✓	✓	-	-	-	-

Becker *et al*. [31], in addition of quality requirements of AAL Solutions, also reports several architecture models with potential utilization for implementing solutions based on the mentioned quality aspects. Nevertheless no functional requirement is specified in any of the mentioned architectural models in Becker *et al*. [31]. As shown in Table 1, there is a straightforward relationship between the quality requirements defined in Becker *et al.* [31] and the functional challenges introduced in this work. 

Considering integrators as part of the system (C1) ensures that only required and proper devices are integrated into the system (heterogeneity and extensibility) keeping down the cost of deployment (affordability). At the same time integrators provide a human interface between assisted people and the system (usability and user experience), ensuring that the system meets all demands of the final user (suitability). The integrators also verify that each device and element of the architecture work properly according to the requirements of the system in a way that the final deployment of the architecture will be able to support most of the required quality features (adaptivity and resource efficiency). In addition, the proper installation of new devices allows the solution to efficiently evolve (C3) (heterogeneity and extensibility) by increasing the capabilities of the system according to the evolution of the assisted users (adaptivity) to cover both new needs but also new skills (usability and user experience). However, such evolution is subject to the available resources to the final users, e.g., cost and the service providers, e.g., human assistance (affordability and resource efficiency).

The improvement of the confidence of the final users is a key factor of acceptance [11]. The user confidence challenge (C5) implies an increase of both understanding of the operations and behavior of the services deployed in the smart space and the control capacity by users. To promote users’ confidence, a common strategy is to avoid adding unfamiliar elements in the smart space and provide computational capacity to the daily objects. This way, since users already have a good understanding of daily objects, they will be more comfortable with this approach (usability and user experience, and suitability). In addition daily objects will be used for current activities in the smart space allowing the reuse of those objects and consequently reducing the final cost (affordability). Finally, user confidence is related with the availability of services, which must be robust and minimize any unexpected results (dependability).

Resilience (C4) guarantees that devices introduced in a home can be used to support users in additional ways than the initially planned (dependability and suitability). This should be done without affecting the behavior of the system or the users’ expectations (usability and users experience). In addition, by addressing this challenge, the amount of devices installed in a home can be reduced because the possibility of using them in different contexts or purposes (affordability and adaptivity).

The evolution of user’s capabilities usually implies the change of the interaction requirements (C2) obtained in the first stages of the solution analysis and design. Due to the evolution of the users’ knowledge, the interaction system can be unaligned with the users’ expectations affecting directly to the user experience or the robustness of the system (dependability and affordability). The proper addressing of this challenge will avoid frustration to users and substantial changes of the solution (usability and user experience, and suitability).

## 4. Definition of the Software Architecture 

The software architecture of a computing system represents the entire structure of the system including the description of all software elements, their properties and the relationship between them [32]. The objective of the software architecture is to define a structured solution that meets all of the functional and operational requirements that are involved in the development of a specific type of service or system [33]. Other details, such as the private details of the elements that make up the architecture and are related to the internal implementation, are not architectural [32] and are outside the scope of this work.

The challenges and requirements that were discussed in the previous section guide the development of the software architecture that is presented in this paper. To define this architecture, we characterize the services that are treated and implemented and their method of deployment in the digital home. We then explain the information exchange model between the entities and describe the modules of the architecture.

### 4.1. Characterization of Sensitive Services

The guidelines that are imposed by assistive and AAL spaces are related to the increase of people’s autonomy and their confidence to enjoy their favorite environments for longer periods of time. To comply with these guidelines, solutions must adapt the environment to people and promote access to basic services for development, social integration and health. A basic service represents the mechanisms that are required to meet the needs of society in matters related to health, education and social participation. These services must also be provided according to criteria of solidarity and social cohesion. ICT provides essential tools for a successful deployment of these services and enables greater efficiency in implementation, closer cooperation between social partners and the satisfaction of the needs of vulnerable groups.

This study considered telemedicine and assistive services as key services to meet the requirements of the proposed smart home platform because of their extensive requirements and challenges. Therefore, services that do not align with these guidelines have not been taken into account in the definition of the smart home platform, although, as discussed later, they may be functionally integrated if they meet the requirements of the communications architecture.

### 4.2. Service Deployment Model

A service deployment model defines the way in which the service is offered to users within an environment. In home environments, it is common for different models, such as those based on personal computers (PCs), to be on appliances or on the network. However, we considered that the digital home should have its own approach to provide the advantages described above. Thus, the model of the digital home must allow for the efficient and dynamic deployment of services based on PCs or networks but also have the reliability and ease of installation of appliance-based models.

**Figure 1:**
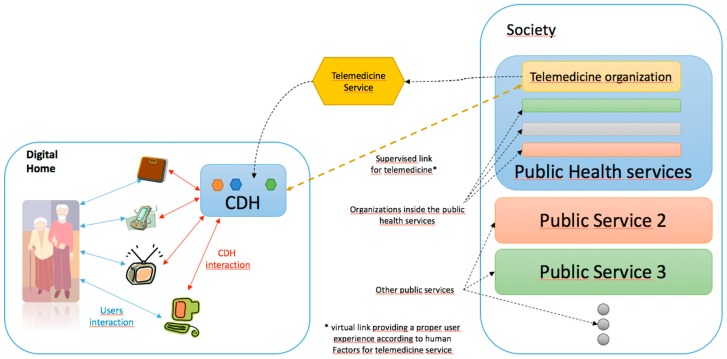
Graphical representation of the deployment of a telemedicine service in the digital home using the developed architecture.

Figure 1 shows the deployment scheme of telemedicine and assistive services in the digital home. The core of the digital home (CDH) is a central element. The CDH is the base element that allows for the creation of the ecosystem of applications within the household. To do this, the CDH ensures:

— The proper integration of all of the devices and applications that are enabled at home;
— The deployment of basic services in the digital home that can make use of embedded devices as well as applications, and;
— Efficient and confident communication with external entities.

**Figure 2:**
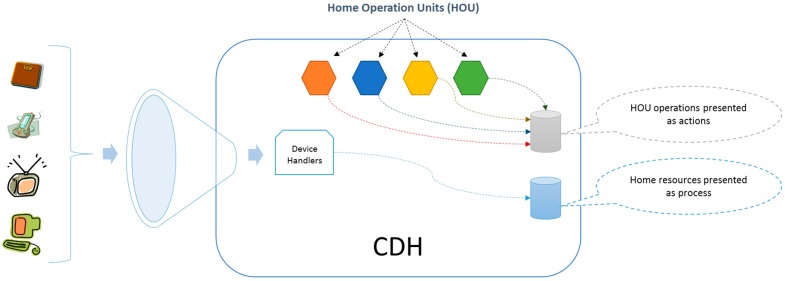
Development of Home Operation Units from real daily objects.

To manage the deployed services, the CDH is responsible for conducting an exploration of existing sensors, actuators and applications in the home, characterizing them according to their specifications and representing them as processes. Then, the Home Operation Units (HOU) deploys each basic service in the CDH as Figure 2 shows. The HOUs implement the house side of a basic service; thus, in the context of this study, we have defined a telemedicine-assistive HOU (it is also possible to deploy other types of HOUs, such as electronic voting and telecare examples). The set of deployed HOUs enriches the capabilities of the home, and these acquired skills are represented in actions. To deploy these actions, the architecture uses household resources, which are represented as processes, so there must be a mapping or a standard agreement between the actions and processes. This mapping can be performed through standardization during the integration or by semantic associations in the case that there is low uncertainty and they are easily verifiable.

The Provider Organization Unit (POU) is defined to complement the HOUs. POUs are within the organizations that provide the service, so they are a representation of the resources and capabilities of the service provider. In this way, the CDH is the entity that manages the life cycle of each HOU and its internal components.

### 4.3. Model Information Exchange between Entities

The information exchange model is a complete definition of the rules of information transmission as well as the language, including syntax and semantics, which is used in the development of telemedicine and assistive services. The full specification of the model is beyond the scope of this paper, but the fundamentals that are needed to understand the architecture are presented.

Because the CDH is designed to offer sensitive services to users with security and acceptance guarantees, the information exchange model must provide the necessary mechanisms to meet all of the requirements related to this type of service. The challenges presented in Section 3 are of special interest, especially challenges 3, 4 and 5, which depend on external entities such as the healthcare center in addition to the homes.

Given these requirements, the information exchange model uses the concept of the Contract-Document (C-D) that is described in [34]. The C-D is an incremental information record that is filled out by the actors involved in a sensitive activity. The actions of all users are reflected in the document with appropriate security safeguards. This allows knowing the status of the transaction at any time for any entity as well as how it was completed.

**Figure 3:**
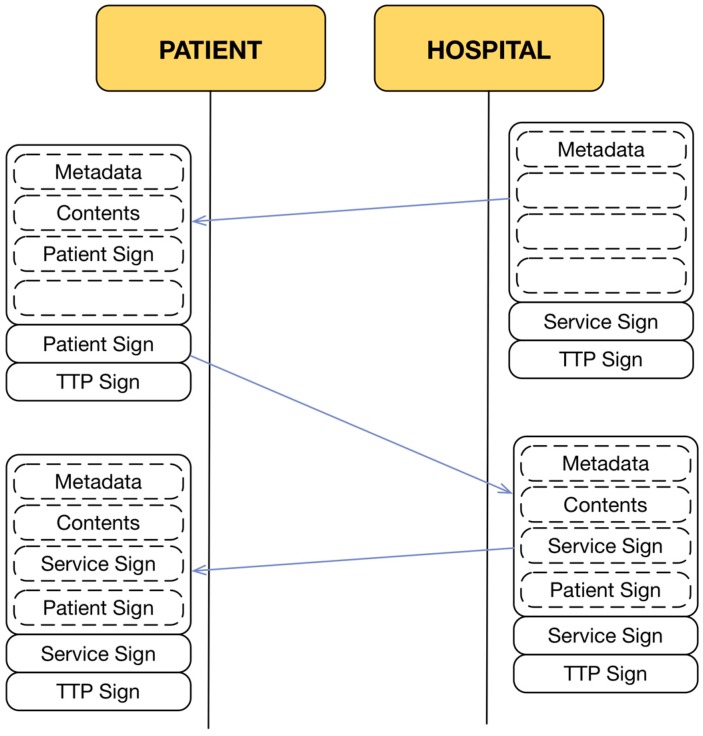
Contract-Document exchange.

At the end of the transaction, both the telemedicine service provider and the patient will obtain a copy of the transaction that was carried out, in which the content of the transaction and the list of actions taken by each entity are clearly reflected (Figure 3). The entities that are involved in this process must show all of this information in a usable and accessible way to achieve a full understanding and correct use by the users. Thus, the interaction between entities is controlled by the document itself through the content that must be refilled by them. However, a C-D is just a generic exchange model that establishes rules for all of the entities that share the same information about a transaction. To implement a specific model for the services discussed in this study, it is necessary to define syntax, semantics and specific implementation rules.

To do this, we used the activity theory and the concept of Activity Centered Design. An activity is composed of a set of operations that are performed to meet the needs of people, and the provision of any basic service will be composed of activities. For example, the telemedicine service could be made up of multiple activities, such as “routine check”, “emergency detection and management”, and “teleconsultation schedule”. All of the exchanges of information between different entities in a given organization should be based on activities, which in turn are governed by the rules reflected in the C-D.

**Figure 4:**
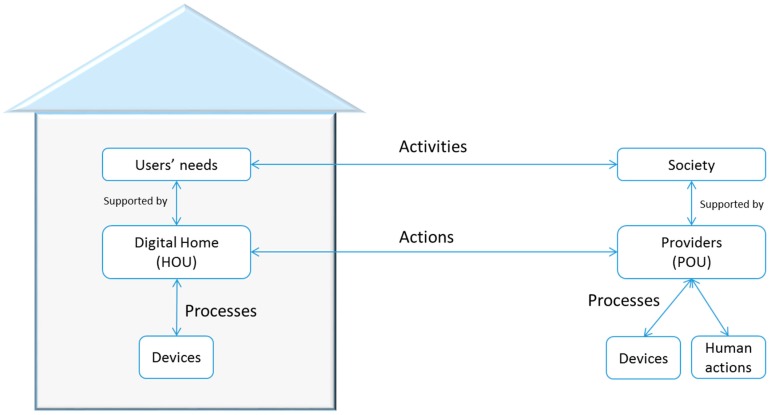
Activity behavior in a telemedicine service deployed in the digital home.

**Figure 5:**
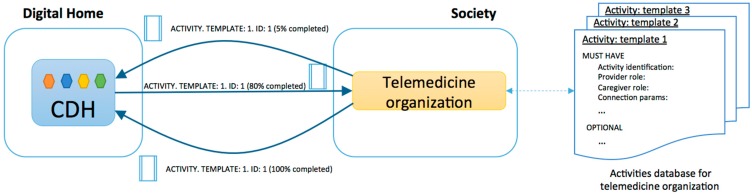
Example of an activity model applied to this study.

Every activity is always guided by goals that satisfy a user need. The activities are divided into actions, which are divided into processes (Figure 4). The actions specify how to execute activities and who will perform each activity. In the case of telemedicine, the actions reflect the clinical procedure that is performed and the organizational health system. The implemented clinical procedure (Figure 5) includes actions that take place within the home, those that are performed by the CDH and the related HOU, and those that are carried out on the premises of the service providers, which are conducted by a POU. 

Finally, the processes develop activities under real conditions that are imposed in a given environment. Depending on the resources that a user has, the actions are divided into different processes. Thus, in two different homes, you might deploy the same activity in different instances if they have adequate HOUs. The two activities have the same actions, irrespective of the housing, so each action to be taken at home will be sent to the appropriate HOU. However, because each home has different configurations, each action is composed of different processes. The action “to obtain confirmation from the user” does not have to be the same in two houses because the context of each can vary significantly; for example, *in a house adapted for blind people, the feedback can never be visual.*

**Figure 6:**
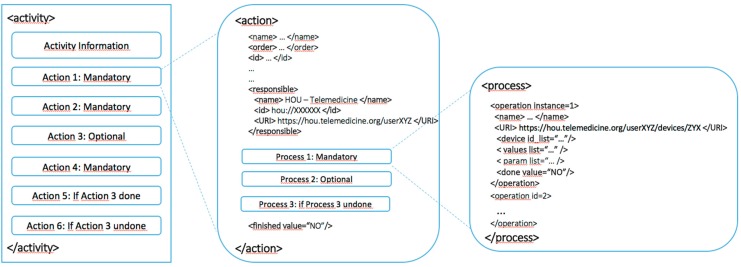
Activity-Action-Process schema.

Figure 6 shows and specifies every significant type of data that defines an activity, such as syntax, labels, required fields, descriptions of the activity’s actions, the conditions for an action to be performed, and the activity life cycle.

This schema shows which entity is in charge of a particular action. Thus, depending on the activity that must be completed, the architecture knows whether the activity should be active at home, in the POU of a service provider, or even in the home of a different user because the identification HOU is unique for the entire application domain. In summary, this information exchange model reflects user activities within the system in the C-D and collects every interaction between users and the system so the users can evaluate the behavior of the system in an understandable way at any time.

### 4.4. Functional Architecture

The software architecture is based on the modules presented above. These modules, except for the HOUs, are generic and occur in all implementations. Because the software architecture presents a design model and the resulting middleware platform, we consider a detailed description of the operation of each HOU to be unnecessary.

Thus, the architecture uses the model defined in Section 4.3 to exchange information at all levels of interaction, both internally between modules and externally between the system and users. The design of activities, actions and associated processes not only defines the behavior of entities when conducting transactions and interactions but also defines the expected behavior by establishing internal communications between modules. This approach is used to determine the advantages offered by the theory of activities to allow the adaptation of this architecture to external changes in functionality or use. In the case of telemedicine, the clinical protocols that are exemplified through activities, actions and processes will be shared by the architecture itself, so there will be no ambiguity or misinterpretation.

**Figure 7:**
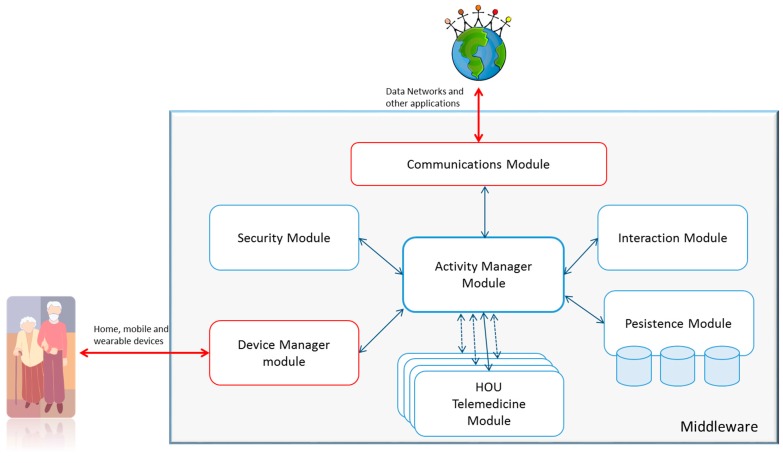
Graphical representation of the architecture’s modules.

As shown in Figure 7, six modules are defined, which must exist in all implementations of the CDH, as well as a set of modules that depend on the services that it can support, which are represented by the HOUs. Each action to be taken within the CDH is referred to a module, as shown in Figure 7. A central module, called the Activity Manager, is responsible for managing the documents that represent the activities to be carried out. Any other module can send processes to this module on its own initiative. From these processes, the Activity Module verifies which actions may be involved and which specific module can perform the action.

Two modules handle outside communications. The Communications Module is responsible for sending and receiving activities through data networks, and the Device Module is responsible for direct communication with the users through compatible devices.

The CDH and its modules will be developed on a middleware that provides the necessary messaging facilities and monitors the status of each of the modules, the lifecycle management, and quality of service. The functionality of each module is described in the following sections.

#### 4.4.1. Activity Manager Module

**Figure 8:**
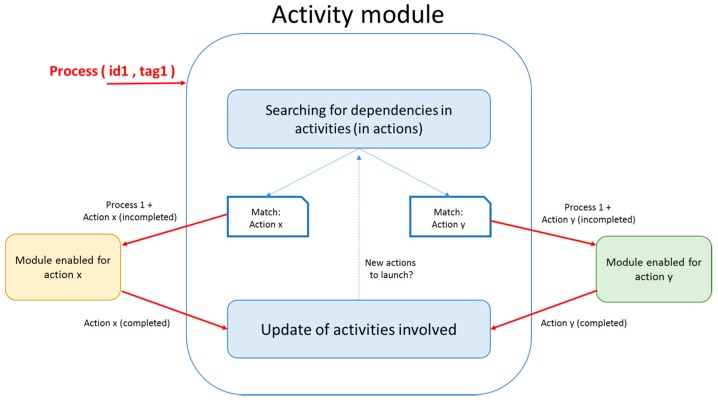
Graphical representation of the Activity module.

As previously described, this module uniquely manages the content of the activities. The activities have an internal language that this module interprets to determine what actions can be performed and what modules can perform them. To perform this procedure, this module receives internal processes from the other modules. These processes are identified inside each action, so the Activity Module can determine that a specific process is related to an action. The Activity Module then sends the received process to the module that is able to complete the action. Once completed, the receiver returns the updated action, and the Activity Module completes the activity with that action. This update can in turn lead to further actions because of interdependencies. Figure 8 shows a graphical representation and internal interactions of this module.

The HOUs perform specific operations of a specific service. To create an adaptive design that is understandable by different actors, each HOU represents its capabilities in the form of actions. These actions are stored in the Persistence Module. Thus, the designers of an activity and the Activity Module can know the capabilities of a house and determine if it is possible to deploy a complete activity. Finally, the service integrator addresses the processes-to-action mapping because they depend on the configuration of the home.

#### 4.4.2. Device Management Module

A device is any element that processes or provides information, such as *physical location, origin, condition, or use*, that extends the concept beyond the sensors and actuators that are offered by the current market for assistance or telemedicine. This means that it should be possible to use everyday objects, such as clothing, furniture or building materials, as devices. To be used in the context of clinical care, these devices must both ensure an optimum level of security, privacy and control and be installed and manufactured by specialized agencies or service providers and integrators.

**Figure 9:**
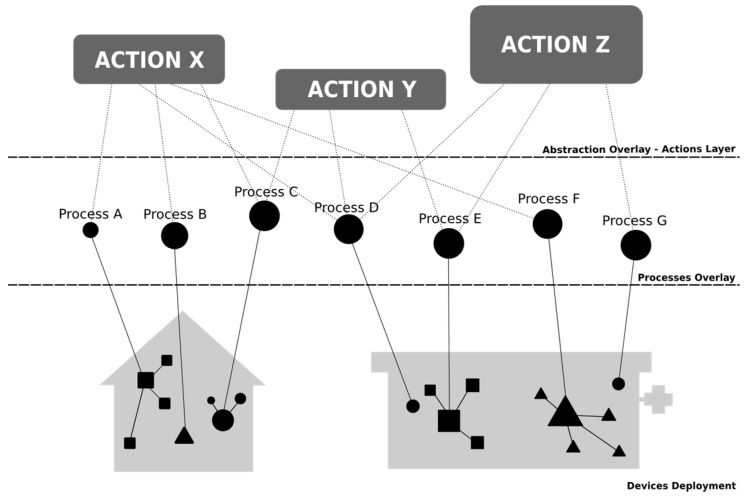
Abstraction layers: model of device-to-process transformation.

This module is in charge of standardizing a set of devices from different manufacturers or entities. Its function is to operate each device on standard processes that are understood by the other modules. This module provides the translation of every device that is compatible with the CDH and HOUs to the processes and their storage in a repository. Thus, this module represents the interface between the architecture and the users’ world. Figure 9 shows how each device is translated to a set of actions in the digital home.

A driver is required for each type of device. Drivers gather the data from the device and build the element of information that is aligned with the process. The relation between the devices and processes is stored in a database that is configured during the integration/installation stage of the CDH deployment. It is then possible to update the database to reflect the changes in the home or users’ devices.

#### 4.4.3. Persistence Module

The information that is generated and managed by the architecture and systems must be tightly housed and stored. The Persistence Module provides a unique address space and model to access all of the information that is handled by the rest of the CDHs. All of the activities, in the current state, will be stored in this module. It also stores the databases that are needed to map the devices to processes or to announce the capabilities of a specific module as actions.

#### 4.4.4. Interaction Module

The ubiquitous systems and highly sophisticated environments that are necessary for the deployment of complex services, such as telemedicine or assistive services, include a large number of items that are usually imperceptible and limit the direct involvement of users. Actively involving humans in the computational processes of these types of environments is difficult without limiting and degrading their potential. However, it is easy to define a global view of these environments by abstractions and determine if the operation and the results are expected. The Interaction Module attempts to define the most appropriate, simple, intuitive and understandable model for each type of user interaction based on the set of activities that the deployed system has defined. Together with the Device Manager Module, this module manages feedback to the users and the communications interfaces that are used for such interactions.

We define a four-actor view of the user ecosystem that characterizes all of the users that are involved in an assistive system from the point of view of smart environments and sensitive services, including:

— End-user;
— Relatives and other informal caregivers;
— Service provider, such as health professionals and healthcare entities; and
— Integrators.

#### 4.4.5. Communications Module

This module manages the communications between remote systems. Telemedicine and assistive solutions have a distributed topology that includes communications between every location that is involved, such as the home and the healthcare center. The communication is based on the exchange of activities using the C-D.

#### 4.4.6. Security Module

Security is a critical aspect, so it is necessary to define security in a way that is comprehensible for all of the stakeholders that are involved in providing the service. Several authors have proposed the implementation of security profiles that are more understandable for users and other stakeholders and also independent of the implemented application or the provided service.

Including security as an independent module of the CDH forces us to define the protection capabilities of the CDH in terms of specific actions. Given their intrinsic nature, such actions would be more intelligible than the descriptions of security mechanisms that are currently used.

The Security Module also manages the users’ credentials and performs data encryption as well as verification and digital signature tasks as required to ensure the correct handling of the service information in terms of both service assurance and privacy.

## 5. Case Study and Verification

The analyzed software architecture has been verified by means of a case study that uses the challenges posed in Section 3 as requirements. The goal of this case study is to demonstrate that the functional description of the architecture is correct. Therefore, the case study presents a partial implementation of the necessary components to show that the proposed approaches meet the identified challenges. The work environment is focused on the digital home, so this implementation uses elements that are associated with this case, such as using *smart daily objects as sensors*.

**Figure 10:**
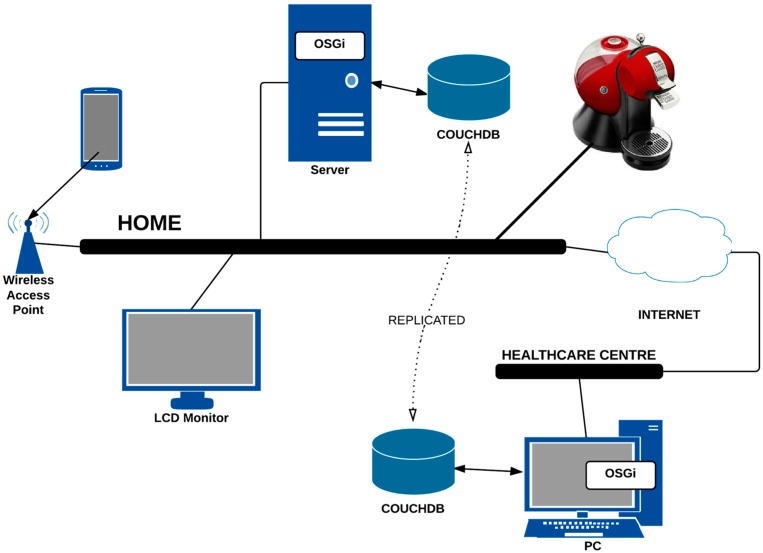
Overall deployment architecture for the case study of assistive services to control the caffeine consumption of hypertensive patients.

The case study presents the deployment of an assistive application whose main objective is to control the consumption of stimulating drinks by patients suffering from hypertension in the digital home. We follow an agile modeling method in which the contents are more important than the representation. The deployment diagram shown in Figure 10 specifies the initial requirements for this development.

### 5.1. Device Installation

First, the elements for the deployment of the defined service are installed. The selected devices are:

(a) A smart coffee maker, which is an example of an everyday object that is endowed with computing capacity, and
(b) Visualization and interaction devices, such as monitors and smartphones.

**Figure 11:**
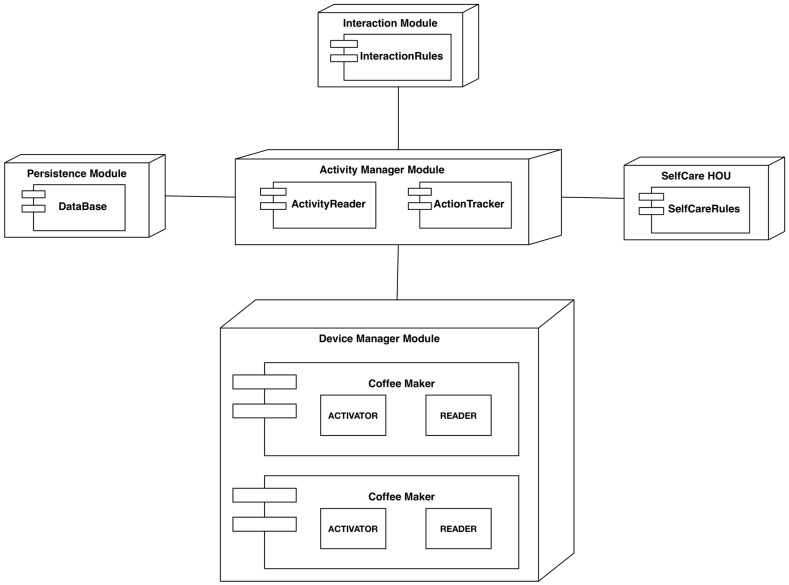
Component diagram.

The installation of these devices is based on the definition of a bundle and the storage of syntactic information following the data model that was defined in Section 4.3. Figure 11 shows the deployment of the software components that are required for this implementation.

**Figure 12:**
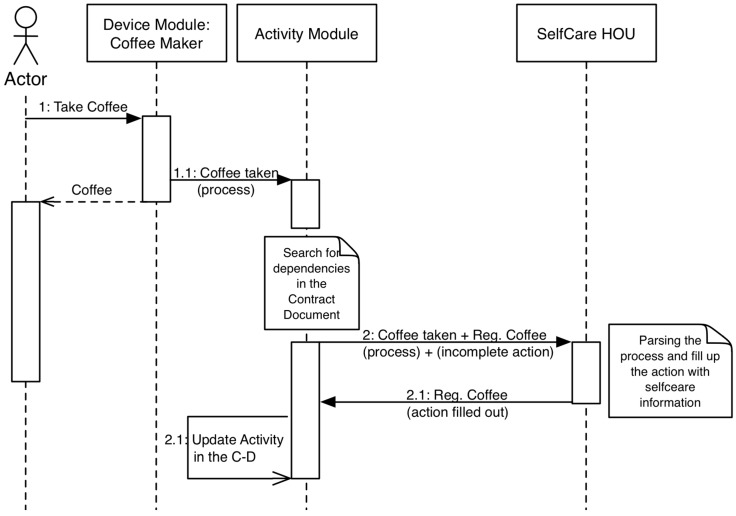
Sequence diagram of the case study.

This example uses a proprietary device, which is the Social Coffee Maker (SCM) [35] from the University of Deusto. This device records the energy consumption of the electric coffee maker and detects the user who performed the action by RFID technology. This information is stored in a document-oriented database (CouchDb). The installation of the SCM into our architecture is performed by the implementation of two bundles: one to access the stored information into CouchDb and one that is related to the action “Register Coffee Consumption”. Figure 12 shows the relationship between the components during the execution of the service.

Access to the information that is recorded by the SCM was implemented using HTTP methods (REST), and the reader class encapsulates that functionality. The information that the SCM stores is related to the energy consumption, but the target of this case study is to offer the action “Register Coffee Consumption”. Thus, the related bundle can filter data to look for this action.

Finally, we developed a bundle to manage the interaction with monitors (or TVs) and smartphones to show relevant information. This bundle provides a REST API that uses a HTTP packet to show its content on a visual interface. We reused an intelligent multi-device user interface service that was previously developed by the authors in [36].

### 5.2. Interaction and User Confidence

Activities define the interactions between the users and the services that were designed with the software architecture. An activity is defined as an XML document within the C-D that specifies a set of actions that the system has to perform. The main activity of this case study is focused on controlling the coffee intake by a user at home. In the case study, we only take into account the first level of definition for an activity, which includes the *activity parameters and the set of actions that are required to deploy this action.* The following Box 1 shows a piece of code that programs this activity:

Box 1 **Implementation of the “Register Caffeine Intake” activity using XML.**


<activity>
	<id>001</id>
  	<definition>Caffeine consumption register.</definition>
  	<others>Other information</others>
  	<actions>
    		<action>Register coffee</action>
  	</actions>
  	<events max_reg="20">
    		<registry>
     	<type>Coffee</type>
     	<date>13/10/13</date>
     	<hour>09:35</hour>
     </registry>
     <registry>
     	<type>Coffee</type>
     	<date>13/10/13</date>
     	<hour>11:21</hour>
     </registry>
  	</events>
</activity>

The action “Show information” is used to isolate the interaction and the application logic. The activity shown in Box 1 makes a record of each consumed coffee that is transparent to the user. This action indicates to the system that the registered information has to be displayed by an interaction device. The system uses monitor screens as display devices to perform this action. The new activity within the C-D is composed of the actions showed in Box 2.

Box 2 **Implementation of a new action to the “Register Caffeine Intake” activity.**


<actions>
     <action>Register coffee</action>
     <action>Show relevant information</action>
</actions>

This model of interaction is implemented through two different bundles. One bundle is associated with the activity whose goal is to process the information that is stored in the XML document, and the other manages the actions. Actions represent simple or complex services that are offered by the installed devices and other applications to the system and to the users.

**Figure 13:**
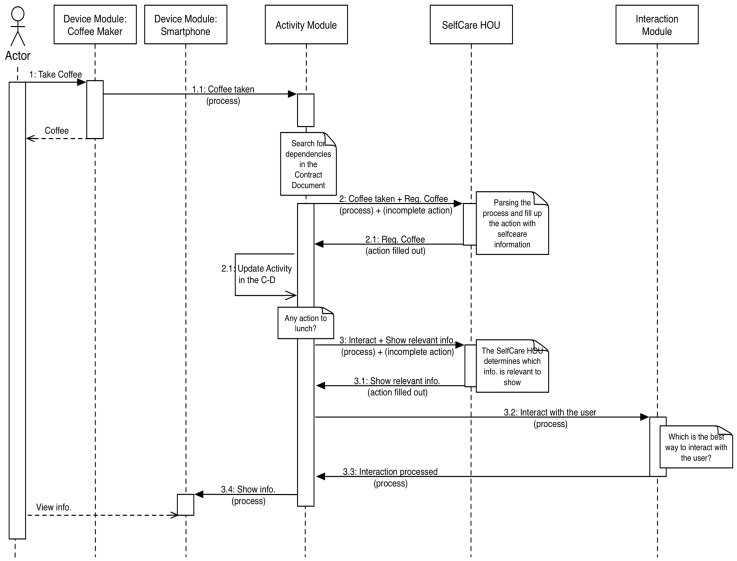
Sequence diagram of the case study updated with the “Show relevant information” action.

The case study uses the OSGi tool ServiceTracker to implement the latter bundle. The aim of this class and the related bundle is to be certain about every service and action that is deployed in the system and provide access to its information. In this way, we know the actions that are registered by the installed devices. Figure 13 shows the new sequence diagram that models the analyzed interaction.

**Figure 14:**
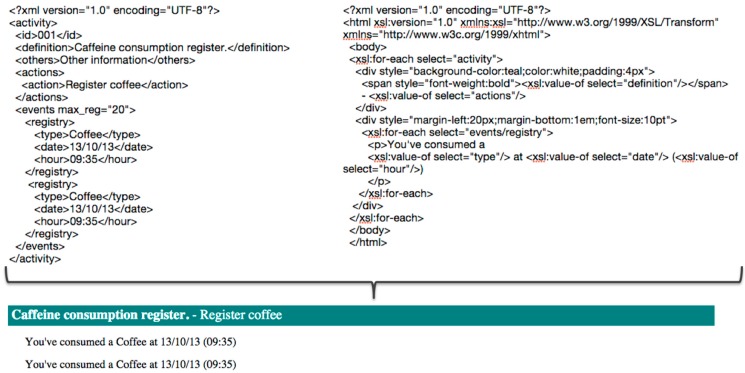
Transformation of an activity in an understandable message.

In summary, this interaction model that is based on activities and C-D takes into account all of the users that are involved in an assistive application. Using XML documents and suitable XSLT, our architecture provides an acceptable level of understanding for all user roles. It is then easy to transform the information that is managed by the system into understandable messages, as is shown in Figure 14.

**Figure 15:**
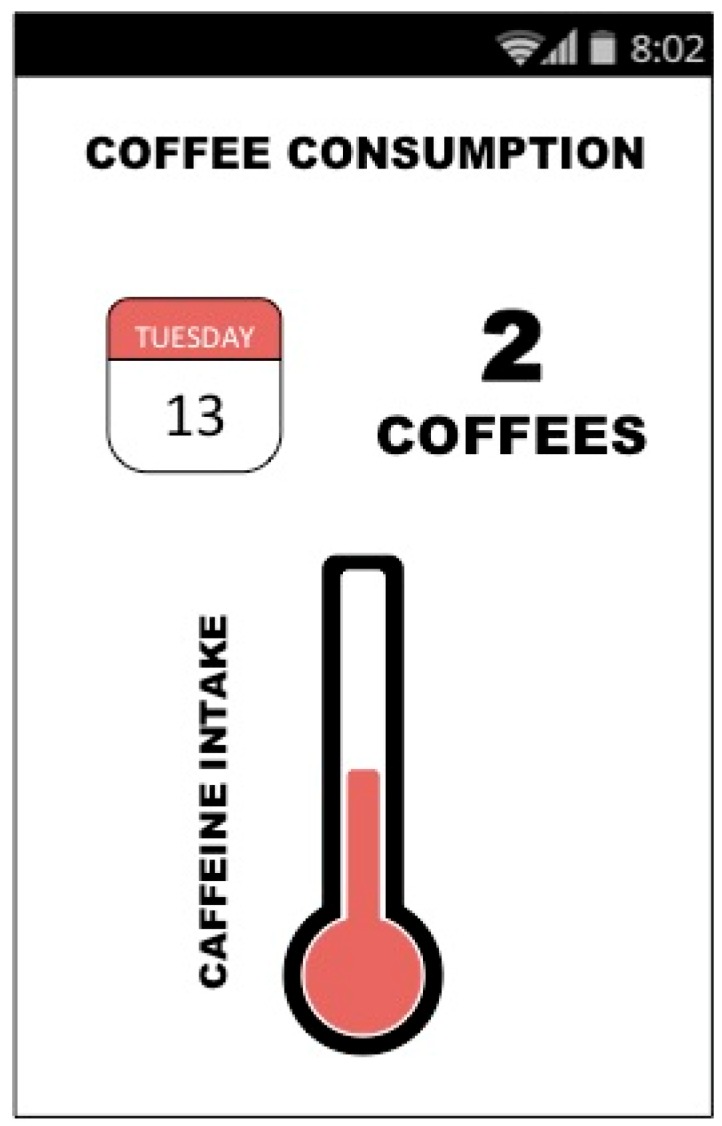
Mobile application to control the user’s caffeine consumption.

We can then create a mobile application to provide feedback to users in an understandable manner, as is shown in Figure 15. 

Finally, the service providers and the final users, including informal caregivers if necessary, define the C-D, while the integrators have a syntactic description of the actions that can be used in the assistive home.

### 5.3. Evolution and Adaptation

The adaptation and evolution of the presented architecture is based on several aspects. From a theoretical point of view, we used a development method that is based on activities called Activity Centered Design (ACD). This method suggests using activities that define the product as analysis and design elements instead of the users’ requirements. Thereby, changes in the user profile, such as user experience or role, are easier to accept and manage.

From a practical point of view, this architecture is guided by the OSGi’s versatility as a tool to effectively manage the lifecycle of the hosted services. OSGi provides mechanisms to evolve the architecture and its applications by including new services or reusing components from other systems without a complex reimplementation.

### 5.4. Resilience

The architecture makes it easy to adapt the system to this new behavior. The RFID tags that are provided by the SCM under the coffee cups are used for this purpose. The SCM assigns registry IDs to each coffee consumption; therefore, with a small improvement (adding a new method) to the associated bundle, it is possible to identify the target user (Box 3).

Box 3 **Example of resilience of the software architecture illustrated by adding a new action to the main activity.**


<actions>
     <action>Register coffee</action>
     <action>Identify Manolo as 55677786</action>
     <action>Show relevant information</action>
</actions>

## 6. Discussion

Software architecture represents a functional model that collects all of the requirements that must be met by a system and defines the necessary communications interfaces to ensure that the elements of the different systems can interact [33]. In a sense, software architecture defines a design template that guides and validates the developments arising from it [32].

From the point of view of the occupants of a home, transforming the space into a smart space raises serious doubts about approval and acceptance. These spaces are often intimate and personal and have emotional connotations that hinder modifications through technology because they seem strange, non-aesthetic and/or complicated to manage. However, the work presented in this paper demonstrates that it is possible to design a software architecture that encompasses technical and theoretical restrictive requirements that allow the design and deployment of sensitive solutions in the digital home. In addition, this software architecture pays special attention in the interactions between the users (the entire ecosystem) and the smart space to promote understanding and control of the deployed systems and therefore the environment. These features have been addressed through the definition of five acceptance challenges, which were defined in Section 3: installing devices (C1), interaction with the user’s home (C2), evolution of the solution (C3), resilience (C4) and user confidence (C5).

The acceptation of the proposed solution has been addressed at two levels: internal (related to the behavior of the entities that are defined in the architecture) and external (the user’s point of view). 

To foster the acceptation at the internal level, the software architecture was developed using patterns based on the activity theory and its related Activity Centered Design model. The resulting architecture forms a robust solution that is focused on activities that simplify its evolution with the users’ experiences. As Norman explains in [14], Activity Centered Design achieves better results in terms of efficiency and adaptability, which makes the system more stable and nurtures the familiarity of the user with the system, which will undoubtedly affect the final acceptance.

*C1: Installing devices.* The installation of new devices is reduced to defining its proper use with a portion of a text file, which is transformed for a specific process by the architecture. The inclusion of the integrator guarantees that the devices that are included and used in the deployed services are well defined and installed. The Device Manager Module and the Interaction Module support the integrators by providing them a guide to adapt the device logic to the system.

*C3: Evolution of the system.* New user requirements are addressed by changes in the activities files that the software architecture can perform. These files are accessible by integrators and service providers to ensure the fitting of the user’s needs.

*C4: Resilience.* Resilience is one of the objectives of the application of activities in the solution design. With other approaches, different components that are part of the solution partially meet objectives that are set previously by the developers (albeit closely agreed upon with users). With an activity-based approach, different components can be used in ways that the user requires. Whether its use contributes to the development of one or more activities will depend on the degree of customization of the system but will not be limited by the design itself. This achieves a greater ability to adapt to changes introduced by the user.

In the case of the acceptation at the external level (C2, C5), the software architecture considers the interaction process to be an independent element. 

*C2: Interaction with the user’s home.* The architecture presents an abstract interaction that is customized to the specific setup of the house. This enables each person to tailor the interaction to their preferences by simply engaging devices for it. 

*C5: User confidence.* The logic of the software architecture is planned in the same way that users would perform their daily activities, which nurtures the understanding of the behavior of the system. Understanding is a key factor of user confidence, but so is the control over the system. For this, the software architecture reflects in the C-D all of the activities that users perform within the smart space. This document attempts to reduce the fears that a user could develop in interacting with sensitive services because they are presented to users in a manner that is readable, understandable and accessible at any time (C5).

The related work that was discussed in Section 2 indicates that there are numerous options for the development and implementation of telemedicine and assistive solutions. A detailed study of the literature about the deployment of telemedicine and assistive services at home shows that the challenges presented in Section 3 must be satisfied to ensure a proper solution. Nevertheless, the research efforts that were reviewed in Section 2 only partially address the identified challenges. The implementation of the case study showed that the proposed software architecture meets the challenges posed in Section 3, which justifies its use as a means for deploying confident telemedicine and assistive services in digital homes. Table 2 compares the presented architecture to the related studies.

In general, the architectures and middleware solutions shown in Table 2 are strongly oriented to programmers and specify the interactions between users and systems by graphical user interfaces. However, the interaction should be designed to create a user experience that is enjoyable for all users regardless of their technical level or capabilities [2]. The feeling of confidence that can be generated during its use and the final acceptance depend strongly on the design and implementation of such interactions.

**Table 2:** Comparison between the proposed architecture and the solutions analyzed in Section 2.

Challenges	ATLAS	S3OiA/WSO2	universAAL	Scherpbier-de Haan *et. al*. [20]	Esser *et al.* [21]	Dabbs *et al.* [22]	Jara *et al.* [23]
1	Only own devices	✓	✓	X	X	X	✓
2	X	✓	X	X	X	✓	X
3	Only in its context	✓	✓	X	X	X	✓
4	X	X	X	X	X	X	X
5	Only for programmers	X	Only for programmers	X	X	X	X

✓: Challenges covered; X: Challenges uncovered.

Only OSGi-based tools guarantee the possibility of evolving and adapting the developed services to new features without degrading the system’s capabilities. From the point of view of design, all of the tested tools follow a user-centered model and are defined to meet specific user requirements. Thus, only installing new devices and implementing new applications can perform the system evolution generated by this model. Some authors believe that User Centered Design is not the correct approach [14] to designing long-lasting systems in which the user experience is expected to change.

## 7. Conclusions

The possibilities for human-computer interaction offered by personal and private smart spaces, such as the digital home, makes them of special interest in fields in which the acceptance of novel technologies is limited and restrictive. The integration of the digital home as a smart space with sensitive solutions can increase user acceptance. However, to be accepted as a valid solution, systems that are based on ubiquitous computing, ambient intelligence and IoT must solve challenges that have not yet been adequately addressed.

This research addresses a current problem of end-user acceptability of smart home technologies by defining a software architecture that meets the typical challenges of sensitive services, such as telemedicine and assistive systems, as well as user acceptance and specifically user confidence. The proposed solution provides a framework to deploy sensitive services into the digital home based on ensuring greater acceptance by every type of users that is involved in these services.

An innovative element of this work is the design methods that are focused on activities to address the development of the software architecture. These methods are widely recognized as key elements for developing user-oriented solutions that allow their evolution with the users’ experiences [14]. The activity theory facilitates the definition of a solid basis for designing a software architecture to specifically address the challenges of evolution, resilience and user confidence.

In addition, implementing the contract document makes it possible to capture the activities that users perform daily and include them in the system. The C-D is a key element of the architecture that allows organizing sensible services according to user behavior; therefore, it provides intrinsic knowledge about how the services are running for the user and provides the user understanding and control. Thus, this research addresses a set of acceptability requirements for sensitive services from both the user interaction and technical points of view, including a novel approach of matching service needs to the smart home’s capabilities via activity templates and the contract-document-exchange communications model. Finally, this paper provides a case study of the software architecture and compares the results with other architectures from the literature to verify that the functional description of the architecture is correct.
